# Role of TREM1 in the sevoflurane‐induced inflammatory activation of microglia in vitro

**DOI:** 10.1002/ibra.12182

**Published:** 2024-09-23

**Authors:** Chunchun Tang, Yuhang Zhu, Dexing Liu, Xue Zheng, Junli Jiang, Wanqiu Yu, Yinan Zhang, Dongqin Chen, Zhaoqiong Zhu

**Affiliations:** ^1^ Department of Anesthesiology Affiliated Hospital of Zunyi Medical University Zunyi China; ^2^ Department of Anesthesiology Maternal and Child Health Care Hospital of Zunyi Zunyi China; ^3^ Early Clinical Research Ward Affiliated Hospital of Zunyi Medical University Zunyi China

**Keywords:** inflammatory activation, microglia, polarization phenotype, sevoflurane, TREM1

## Abstract

Perioperative neurocognitive disorders (PNDs) are one of the most common complications in perioperative patients, and neuroinflammatory reaction mediated by microglia plays a key role in their formation, but the underlying mechanism remains unknown. Given that the triggering receptor expressed on myeloid cells 1 (TREM1) is a key regulator of inflammation, this study aimed to observe the role of TREM1 on the sevoflurane‐induced inflammatory activation in microglia. BV2 microglia were subjected to varying sevoflurane concentrations and durations to assess their viability using CCK8 and the expression of TREM1, iNOS, and ARG using enzyme‐linked immunosorbent assays. Additionally, TREM1 knockdown lentivirus was employed to examine its impacts on microglia response to sevoflurane and altered expression of inflammatory markers, IL‐1β, TNF‐α, TGF‐β, IL‐10, iNOS, and ARG, as detected using qRT‐PCR and immunofluorescence for INOS/Iba‐1 and ARG/Iba‐1. Our findings underscore the potent inflammatory activation induced by prolonged, high‐concentration sevoflurane exposure on microglia. We highlight the potential role of TREM1 as a modulator of microglial polarization and a potential target for the treatment and prevention of sevoflurane‐induced PNDs.

## Graphical abstract

Perioperative neurocognitive disorders (PNDs) are one of the most common complications in perioperative patients, and neuroinflammatory reaction mediated by microglia plays a key role in their formation. Triggering receptor expressed on myeloid cells 1 (TREM1) is a key regulator of inflammation. This study aimed to observe the role of TREM1 in the inflammatory activation of sevoflurane‐induced microglia. Our findings underscore the potent inflammatory activation induced by prolonged, high‐concentration sevoflurane exposure on microglia. We highlight the potential role of TREM1 as a modulator of microglial polarization and a potential target for the treatment and prevention of sevoflurane‐induced PNDs.

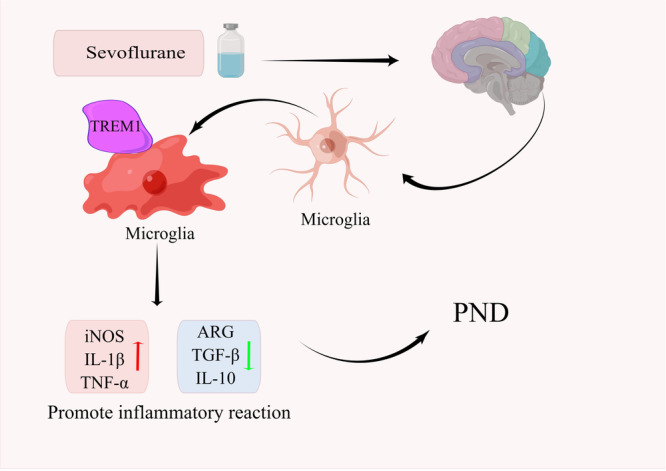

## 1 INTRODUCTION

Perioperative cognitive disorders (PNDs) are common complications during the perioperative period, which not only prolong hospital stay and increase the cost of treatment but also elevate the risk of other related complications and postoperative mortality.^1^, ^2^ Currently, sevoflurane is one of the most commonly used inhaled general anesthetics in clinical practice. Studies have increasingly demonstrated its potential for causing brain neuron damage^3^, ^4^ and impairing the learning and memory functions of postoperative patients. Sevoflurane‐induced neurotoxicity (SIN) is closely related to PNDs.^5^ While the neuroinflammatory reaction mediated by microglia plays a key role in the occurrence and development of SIN,^6^, ^7^, ^8^ its exact mechanism remains unclear. Currently, there is a paucity of relevant literature, both local and international, that describes sufficient data between specific concentration and intervention duration of sevoflurane in microglia‐related research.

Although studies have found that triggering receptor expressed on myeloid cells 1 (TREM1) is an inflammatory amplifier that plays a key role in neuroinflammatory‐related diseases, such as ischemic stroke and subarachnoid hemorrhage (SAH),^9^, ^10^ TREM1 has not been studied in the context of microglia‐mediated SIN.

**Figure 1:**
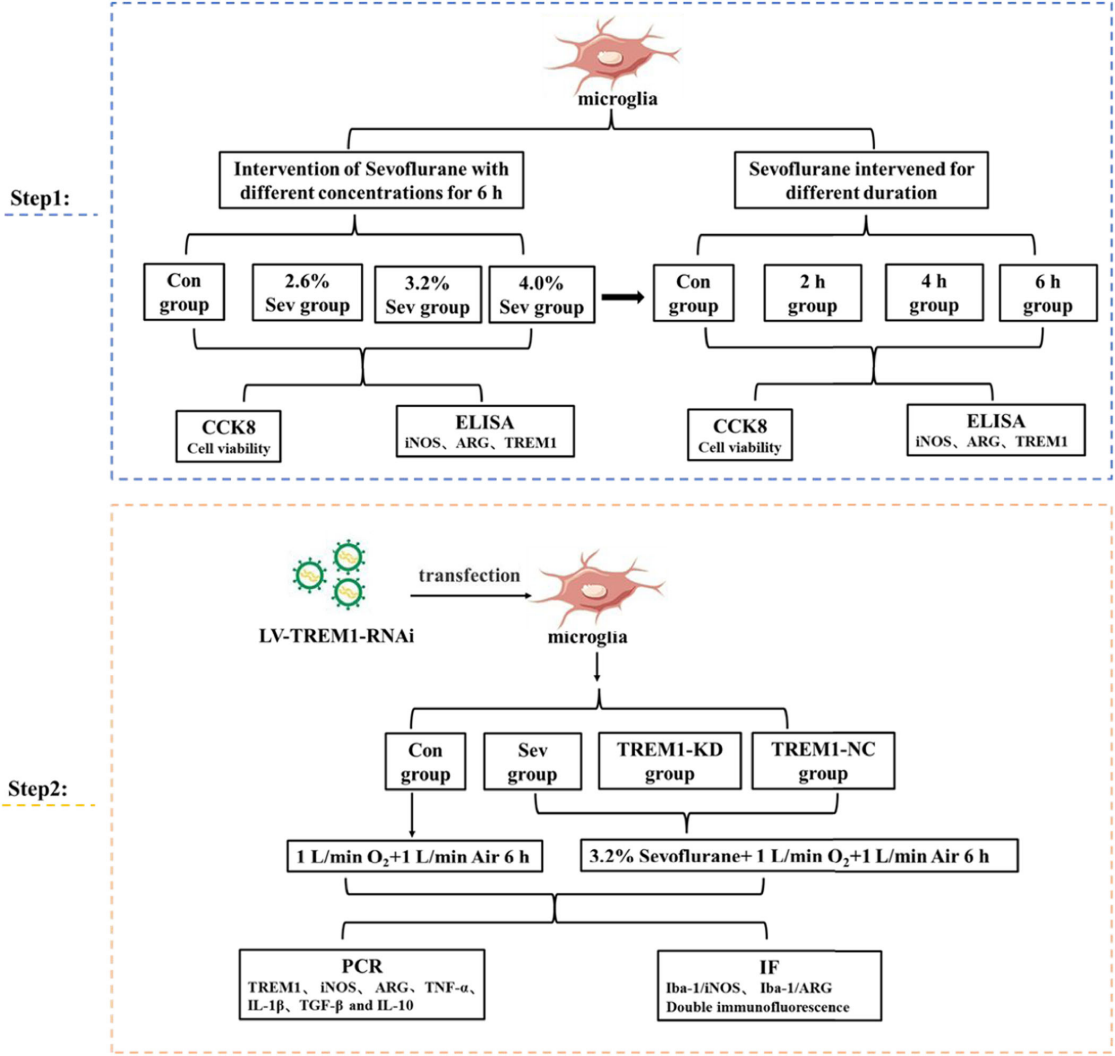
The schedule of the experiment. [Color figure can be viewed at wileyonlinelibrary.com]

Hence, this study investigated the impact of sevoflurane on microglia, including its dose‐effect and time‐effect correlation by using an optimal system in vitro, and subsequently explored the role of TREM1 in sevoflurane‐mediated microglia activation to provide a novel therapeutic target for the prevention and treatment of SIN. The design of this study is illustrated in Figure 1.

## 2 MATERIALS AND METHODS

### 2.1 Experimental subject

BV2 microglia cells were procured from Wuhan Punosai Life Science and Technology Co., Ltd. (No.: CL‐0493). The cells were cultured under conditions of 37 ± 0.5°C, 5% CO_2_, 95% air, and saturated humidity.

### 2.2 Experimental grouping

After BV2 microglia were cultured to the logarithmic growth stage, they were grouped according to different research purposes for subsequent experiments.

#### 2.2.1 Effects of different concentrations of sevoflurane on microglia

This part of the experiment was divided into four groups: the control (Con) group, 2.6% sevoflurane (Sev) 6 h group, 3.2% Sev 6 h group, and 4.0% Sev 6 h group. Table 1 describes the grouping and processing.

**Table 1:** Experimental grouping of dose‐effect relationship of sevoflurane on BV2 microglia.

Group	Intervention measure
Con	Without any intervention measures
2.6% Sev 6 h	Exposure to 2.6% sevoflurane for 6 h
3.2% Sev 6 h	Exposure to 3.2% sevoflurane for 6 h
4.0% Sev 6 h	Exposure to 4.0% sevoflurane for 6 h

Abbreviations: Con, control; Sev, sevoflurane.

#### 2.2.2 Effects of sevoflurane intervention on microglia in different durations

This part of the experiment was divided into four groups: the Con group, 3.2% Sev 2 h group, 3.2% Sev 4 h group, and 3.2% Sev 6 h group. Table 2 describes the grouping and processing.

**Table 2:** Experimental grouping of the time‐effect relationship of sevoflurane on BV2 microglia.

Group	Intervention measure
Con	Without any intervention measures
3.2% Sev 2 h	Exposure to 3.2% sevoflurane for 2 h
3.2% Sev 4 h	Exposure to 3.2% sevoflurane for 4 h
3.2% Sev 6 h	Exposure to 3.2% sevoflurane for 6 h

Abbreviations: Con, control; Sev, sevoflurane.

Through the above (2.2.1 and 2.2.2) studies, the intervention concentration and duration of sevoflurane were selected for the follow‐up experiments.

#### 2.2.3 Role of TREM1 in the inflammatory activation of sevoflurane‐induced microglia

This part of the experiment was divided into four groups: the Con group, Sev group, TREM1‐KD group, and TREM1‐NC group. Table 3 describes the grouping and processing.

**Table 3:** Role of TREM1 in sevoflurane‐induced inflammatory activation of microglia.

Group	Intervention measure
Con	Without any intervention measures
Sev	Sevoflurane exposure
TREM1‐KD	Sevoflurane exposure after transfection of lentivirus with TREM1 knockdown
TREM1‐NC	Sevoflurane exposure after transfection of negative control lentivirus

Abbreviations: Con, control; Sev, sevoflurane; TREM1, triggering receptor expressed on myeloid cells 1.

### 2.3 Cell culture

BV2 cells were cultured in Minimum Essential Medium containing 10% fetal bovine serum, 0.5% penicillin (10,000 U/mL), and 0.5% streptomycin (10,000 μg/mL).

### 2.4 Sevoflurane intervention

The method of cell exposure has been described by Xie et al.^11^ When the cell growth density reached 80%−90%, cells with the same growth generation were selected and placed in a homemade sevoflurane cell incubator. The carrier gas was CO_2_ 0.1 L/min + Air 1.9 L/min. The concentration of sevoflurane was determined according to the grouping, and the temperature was maintained at 37 ± 0.5°C. According to the experimental groups, the cells were exposed to sevoflurane at different concentrations and for different durations. After the exposure, the cells were collected for subsequent experiments.

### 2.5 Detection of cell viability

The cell suspension was inoculated into 96‐well plates with 8 × 10^3^ cells per well. After adding a complete medium to make the volume of each well reach 100 μL, the cells were cultured for 24 h. Subsequently, the cells in each group were immediately added to 10 μL of CCK8 (Solarbio) solution and placed in an incubator for 3 h, after which the absorbance was detected at 450 nm using an enzyme‐labeled instrument. Cell viability = (experimental group − blank group)/(control group − blank group) × 100%.

### 2.6 LV‐TREM1‐RNAi‐transfected BV2 microglia

LV‐TREM1‐RNAi was synthesized by Shanghai Genechem Co., Ltd. Cell suspension at a density of 5 × 10^4^ cells/mL was prepared with a complete culture medium and the corresponding cells were inoculated into the culture plate (the cell inoculation volume was determined according to the cell culture container). The cells were cultured at 37°C for 16−24 h until the confluence of cells reached 20%−30%. The cells were then matched with multiplicity of infection and virus titer, which were determined in prior experiments. Subsequently, the cells were cultured at 37°C for 12−16 h and then changed to complete medium to continue culture. After incubating lentivirus‐infected BV2 microglia for 72 h, the cell fusion degree reached 70%−80%. The cells were continuously cultured in a culture solution containing purinase with a proper concentration and after screening for 48 h. The concentration of purinase was reduced to the maintenance concentration (one‐half to one‐quarter of the screening concentration), and infected cells were screened and expanded for subsequent experiments.

### 2.7 ELISA

An enzyme‐linked immunosorbent assay (ELISA) kit was purchased from Shanghai Jianglai Biotechnology. Cells from different groups were collected, and cell suspension was prepared. Cell concentration was adjusted to around 1 million cells per milliliter. The cell suspension was frozen at −20°C for 30 min and unfrozen at 37°C. This process was repeated 3−5 times. Suspension was centrifuged at 3,000 rpm, 4°C, and for 20 min. After centrifugation, the supernatant was collected as the sample to be tested. Then, 100 μL of standard substance and sample to be tested was added with different concentrations into the enzyme‐labeled plate, incubated at 37°C for 1 h, and the liquid was discarded. Subsequently, 100 μL of biotinylated antibody working solution was added to each well, incubated at 37°C for 1 h, and the liquid was discarded. Furthermore, washing liquid was added, which was allowed to stand for 1 min and was discarded subsequently. Enzyme conjugate working solution (100 μL) was added to each well and incubated at 37°C for 30 min, the solution was discarded, and the plate was washed five times. The substrate (90 μL) was added and incubated at 37°C in the dark for 15 min. Stop solution (50 μL) was added to each well, and the optical density (OD) of each well was immediately measured at a wavelength of 450 nm. The linear regression curve of the standard was drawn, and the concentration value of each sample was calculated using the curve equation.

### 2.8 Immunofluorescence staining

The slides of cells went through the steps of fixation, permeability, and sealing. After incubating the primary antibodies Iba‐1 (1:100; Mouse polyclonal antibody, Abcam), iNOS (1:40; Rabbit polyclonal antibody, Novus), and ARG (1:200; Rabbit polyclonal antibody, Protetech) 4°C overnight, each slide was washed three times with phosphate‐buffered saline for 5 min per wash. The corresponding secondary antibody (1:1000, Alexa Fluor 647 labeled sheep anti‐mouse IgG, Cellsignal; 1:500, Alexa Fluor 555 labeled goat anti‐rabbit IgG, Solarbio) was added dropwise and incubated at room temperature for 60 min in the dark. After DAPI (Solarbio) stained the nucleus, the seal was observed and photographed under confocal microscope (Zeiss, Wetzlar). The confocal scanning parameters were as follows Table 4.

**Table 4:** The confocal scanning parameters.

	Channel name	AF‐647	AF‐555
iNOS	Pinhole	70 μm	50 μm
	Detector gain	800 V	750 V
ARG	Pinhole	80 μm	120 μm
	Detector gain	770 V	700 V

### 2.9 qRT‐PCR

Total RNA was isolated from cells in different groups by RNAiso plus (TaKaRa). The quality of RNA was assessed based on the OD ratio 260/280, and samples with OD 260/280 > 1.8 were included. Reverse transcription to generate cDNA was performed using reverse transcription kit synthesis (Servicebio) at 37°C for 15 min, followed by denaturation at 85°C for 5 s and incubation at 4°C indefinitely. Quantitative PCR (Servicebio) involved pre‐denaturation at 95°C for 30 s; denaturation at 95°C for 15 s; annealing/extension at 60°C for 30 s, for a total of 40 cycles; and melting at 65°C−95°C, with fluorescence signal every time the temperature increased by 0.5°C. *GAPDH* was used as an internal reference for standardization, and the experimental results were analyzed using the 2^−^^ΔΔCt^ method. The primer sequences are shown in Table 5.

**Table 5:** Primer sequence of RT‐PCR.

Gene		Sequence（5′‐3′)
GAPDH	F:	CCTCGTCCCGTAGACAAAATG
	R:	TGAGGTCAATGAAGGGGTCGT
IL‐1β	F:	GCATCCAGCTTCAAATCTCGC
	R:	TGTTCATCTCGGAGCCTGTAGTG
TNF‐α	F:	CCCTCACACTCACAAACCACC
	R:	CTTTGAGATCCATGCCGTTG
TGF‐β	F:	TAATGGTGGACCGCAACAAC
	R:	CCACATGTTGCTCCACACTTGAT
IL‐10	F:	AATAAGCTCCAAGACCAAGGTGT
	R:	CATCATGTATGCTTCTATGCAGTTG
iNOS	F:	CAACAGGAACCTACCAGCTCACT
	R:	AGCCTGAAGTCATGTTTGCCG
ARG	F:	CCGTCTATGGCGTGTCTCCTAT
	R:	CTCCAGGGTACACACACCTAGCA
TREM1	F:	GTTGTGCTCTTCCATCCTGTCC
	R:	GTCCCATTTATGATAGTGACTCCAAGA

### 2.10 Statistical analysis

SPSS 18.0 (IBM) was used for analysis, and the results were expressed as mean ± standard deviation. One‐way analysis of variance was used to test the differences among the groups. When the differences were statistically significant, pairwise comparisons were made, the least significant differences test was performed when the variance was homogeneous, and Dunnett's T3 test was performed when the variance was uneven. *p* < 0.05 was determined to be statistically significant.

## 3 RESULTS

### 3.1 Effect of sevoflurane on microglia and its dose‐effect and time‐effect correlations

#### 3.1.1 Comparison of microglia viability, TREM1, iNOS, and ARG among different groups after sevoflurane exposure

**Figure 2:**
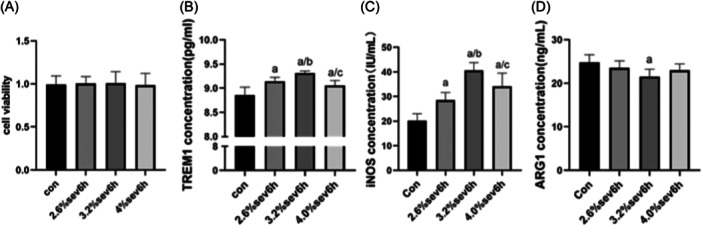
Effect of sevoflurane on BV2 microglia and its dose‐effect correlation. (A) Effects of sevoflurane exposure with different concentrations on the cell viability of BV2 microglia. (B−D) Enzyme‐linked immunosorbent assays were used to detect the expression of TREM1, iNOS, and ARG in BV2 microglia after sevoflurane exposure at different concentrations, respectively. Con, control; Sev, sevoflurane. *n* = 6. Compared with Con group, ^a^ *p* < 0.05; compared with 2.6% Sev group, ^b^ *p* < 0.05; compared with 3.2% Sev group, ^c^ *p* < 0.05. TREM1, triggering receptor expressed on myeloid cells 1.

After sevoflurane exposure to different concentrations for 6 h, compared with the Con group, there was no significant change in microglia viability among sevoflurane groups with different concentrations (*p* > 0.05, Figure 2A).

However, after microglia exposure to sevoflurane at different concentrations for 6 h, the expression of TREM1 in the microglia of sevoflurane groups with different concentrations was higher than that in the Con group (*p* < 0.05), whereas the expression of TREM1 in Con, 2.6% Sev, and 4.0% Sev groups was lower than that in the 3.2% Sev group (*p* < 0.05, Figure 2B). Similarly, the expression of iNOS in microglia of sevoflurane groups with different concentrations was higher than that of the Con group (*p* < 0.05). Among them, the 3.2% Sev group had the most pronounced increase, which was statistically significant compared with the 2.6% Sev and 4.0% Sev groups (*p* < 0.05, Figure 2C).

As for the ARG, it shown that the expression of ARG in the 3.2% Sev group was lower than that in the Con group (*p* < 0.05). Although ARG in the 2.6% Sev and 4.0% Sev groups showed a downward trend compared with Con group, there was no significant difference between the two groups (*p* > 0.05, Figure 2D).

#### 3.1.2 Comparison of microglia vitality, TREM1, iNOS, and ARG after sevoflurane exposure for different periods of time

**Figure 3:**
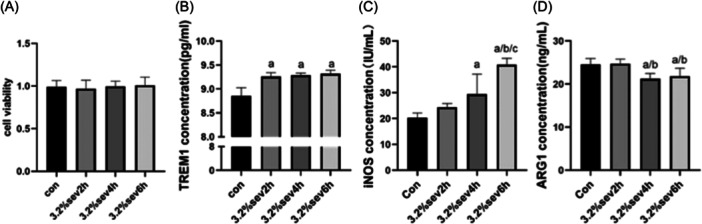
Effect of sevoflurane on BV2 microglia and its time‐effect correlation. (A) Effects of sevoflurane exposure for different durations on the viability of BV2 microglia. (B−D) Enzyme‐linked immunosorbent assays were used to detect the expression of TREM1, iNOS, and ARG in BV2 microglia after sevoflurane exposure for different duration. Con, control; Sev, sevoflurane. *n* = 6. Compared with Con group, ^a^ *p* < 0.05; compared with 3.2% Sev 2 h group, ^b^ *p* < 0.05; compared with 3.2% Sev 4 h group, ^c^ *p* < 0.05. TREM1, triggering receptor expressed on myeloid cells 1.

After microglia exposure to 3.2% sevoflurane for different durations, compared with the Con group, there was no significant change in microglia viability between the sevoflurane groups (*p* > 0.05, Figure 3A).

However, it is worth noting that, after microglia exposure to sevoflurane at 3.2% concentration for different time periods, the expression of TREM1 in microglia of the sevoflurane group was higher than that of the Con group (*p* < 0.05, Figure 3B). In addition, the expression of iNOS in microglia exposed to sevoflurane for 4 and 6 h was higher than that in the Con group (*p* < 0.05). Among them, exposure for 6 h was the most obvious, and the difference was also statistically significant compared with the exposure for 2 and 4 h (*p* < 0.05, Figure 3C). However, after microglia exposure to sevoflurane at 3.2% concentration for different durations, ARG in microglia of sevoflurane‐exposed groups for 4 and 6 h was lower than that of the Con group and sevoflurane‐exposed group for 2 h (*p* < 0.05, Figure 3D).

### 3.2 TREM1 knockdown reduces the expression of inflammatory factors in microglia after sevoflurane exposure

Based on the effect of sevoflurane on microglia and the results of its dose‐effect and time‐effect correlation, the concentration of sevoflurane intervention was set at 3.2% and the intervention duration was set at 6 h. After sevoflurane intervention, cells were collected for subsequent detection.

#### 3.2.1 TNF‐α

**Figure 4:**
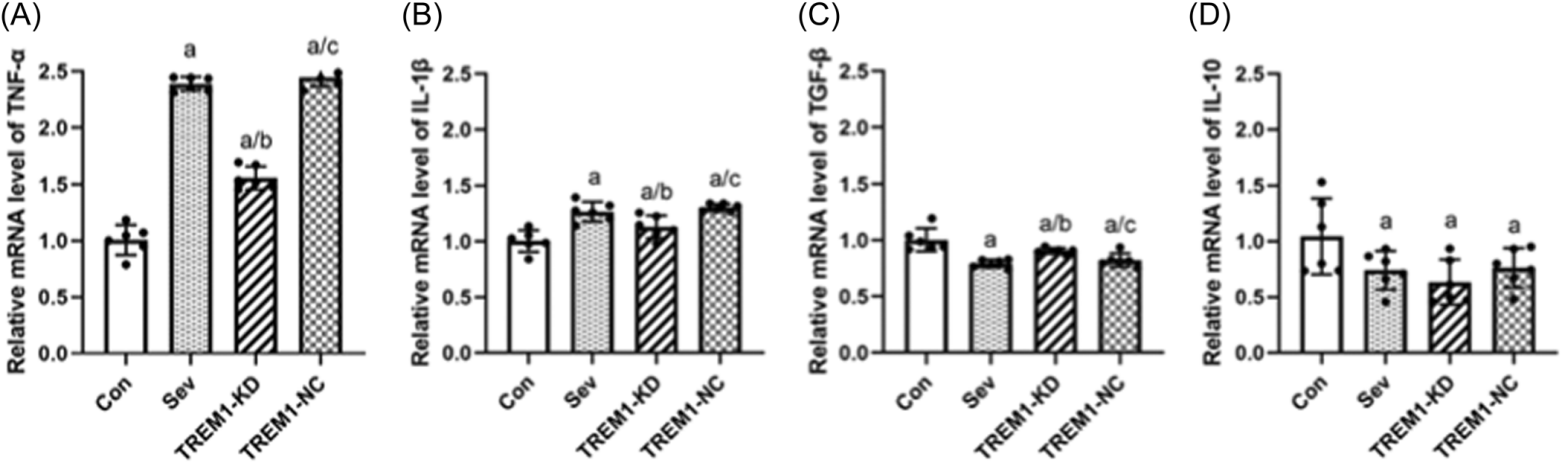
Effect of triggering receptor expressed on myeloid cells 1 (TREM1) on mRNA expression of inflammatory factors in microglia after sevoflurane exposure. (A−D) qRT‐PCR was used to detect TNF‐a, IL‐1β, TGF‐β, and IL‐10. Con, control; Sev, sevoflurane. *n* = 6. Compared with the Con group, ^a^ *p* < 0.05; compared with the Sev group, ^b^ *p* < 0.05; compared with the TREM‐KD group, ^c^ *p* < 0.05.

After 6 h exposure of microglia to 3.2% sevoflurane, TNF‐α expression in the Sev, TREM1‐KD, and TREM1‐NC groups was higher than that in the Con group (*p* < 0.05). TNF‐α expression in the TREM1‐KD group was lower than that in the Sev group (*p* < 0.05), and the TREM1‐NC group(*p* < 0.05, Figure 4A).

#### 3.2.2 IL‐1β

After 6 h exposure of microglia to 3.2% sevoflurane, the expression of IL‐1β in the Sev, TREM1‐KD, and TREM1‐NC groups was higher than that in the Con group (*p* < 0.05). IL‐1β expression in the TREM1‐KD group was lower than that in the Sev group (*p* < 0.05) and the TREM1‐NC group (*p* < 0.05, Figure 4B).

#### 3.2.3 TGF‐β

After 6 h exposure of microglia to 3.2% sevoflurane, TGF‐β expression in the Sev, TREM1‐KD, and TREM1‐NC groups was lower than that in the Con group (*p* < 0.05). However, TGF‐β expression in the TREM1‐KD group was higher than that in the Sev group (*p* < 0.05) and TREM1‐NC group (*p*< 0.05, Figure 4C).

#### 3.2.4 IL‐10

After 6 h exposure of microglia to 3.2% sevoflurane, IL‐10 expression in the Sev, TREM1‐KD, and TREM1‐NC groups was lower than that in the Con group (*p* < 0.05, Figure 4D).

### 3.3 TREM1 regulates the polarization phenotype of microglia after sevoflurane exposure

#### 3.3.1 TREM1

**Figure 5:**
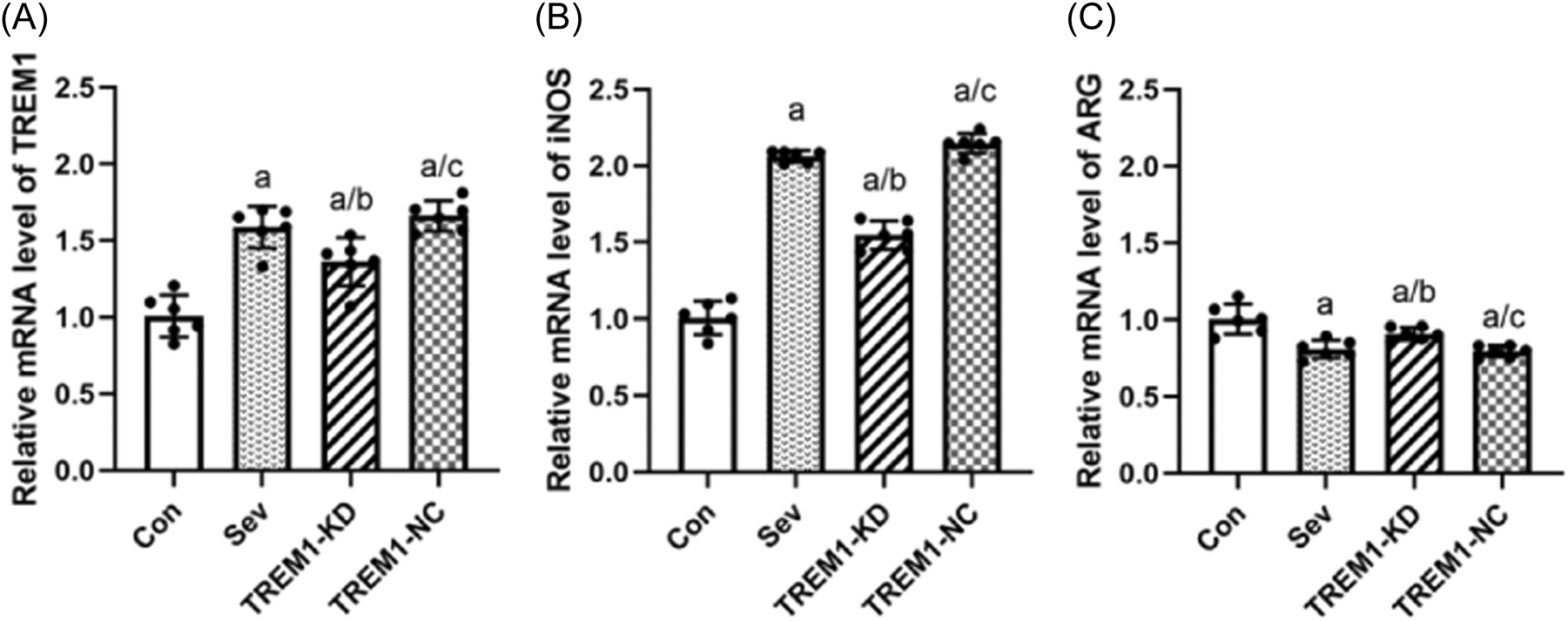
Effect of triggering receptor expressed on myeloid cells 1 (TREM1) on polarization phenotype of microglia after sevoflurane exposure. (A−C) qRT‐PCR was used to detect the expression of TREM1, iNOS, and ARG. *n* = 6. Compared with the Con group, ^a^ *p* < 0.05; compared with the Sev group, ^b^ *p* < 0.05; and compared with the TREM‐KD group, ^c^ *p* < 0.05.

After 6 h exposure of microglia to 3.2% sevoflurane, TREM1 expression in the Sev, TREM1‐KD, and TREM1‐NC groups was higher than that in the Con group (*p* < 0.05). TREM1 expression in the TREM1‐KD group was lower than that in the Sev group (*p* < 0.05) and TREM1‐NC group (*p* < 0.05, Figure 5A).

#### 3.3.2 iNOS

After 6 h exposure of microglia to 3.2% sevoflurane, iNOS expression in the Sev, TREM1‐KD, and TREM1‐NC groups was higher than that in the Con group (*p* < 0.05). iNOS expression in the TREM1‐KD group was lower than that in the Sev group (*p* < 0.05) and the TREM1‐NC group (*p* < 0.05, Figure 5B).

#### 3.3.3 ARG

After 6 h exposure of microglia to 3.2% sevoflurane, ARG expression in the Sev, TREM1‐KD, and TREM1‐NC groups was lower than that in the Con group (*p* < 0.05). ARG expression in the TREM1‐KD group was higher than that in the Sev group (*p* < 0.05) and TREM1‐NC group (*p* < 0.05, Figure 5C).

#### 3.3.4 Results of immunofluorescence co‐localization of iNOS/Iba‐1 and ARG/Iba‐1

**Figure 6:**
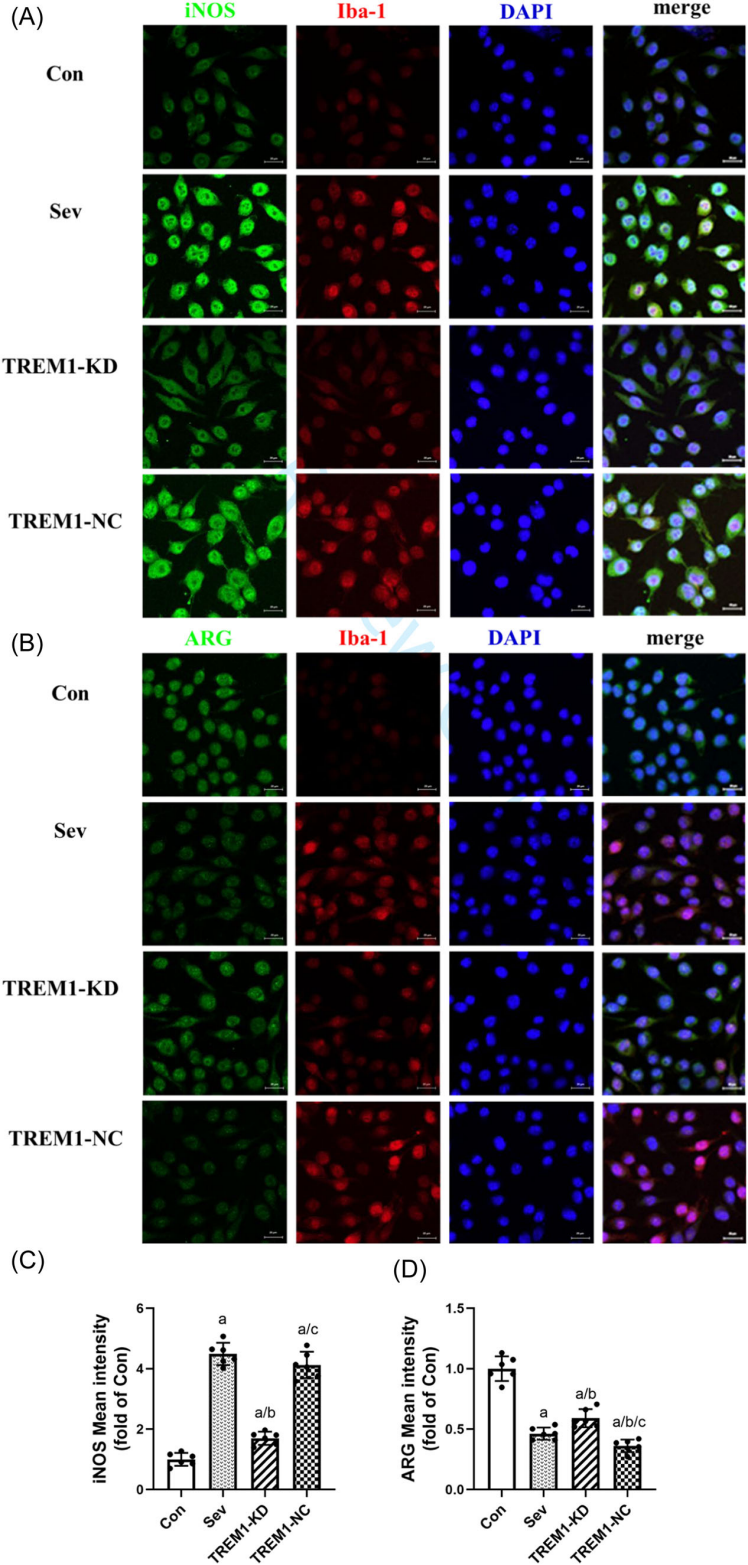
Immunofluorescence shows that triggering receptor expressed on myeloid cells 1 (TREM1) regulates the polarized phenotype of microglia after sevoflurane exposure. (A, B) After sevoflurane exposure, the results of double immunofluorescence of microglia Iba‐1 (red)/iNOS (green) and Iba‐1 (red)/ARG (green). (C, D) Fluorescence intensity levels of iNOS and ARG. *n* = 6. Compared with the Con group, ^a^ *p* < 0.05; compared with the Sev group, ^b^ *p* < 0.05; compared with the TREM‐KD group, ^c^ *p* < 0.05. Scale bar = 20 μm. TREM1, triggering receptor expressed on myeloid cells 1. [Color figure can be viewed at wileyonlinelibrary.com]

To further confirm the role of TREM1 in the variation in polarization phenotype of microglia exposed to sevoflurane, M1 and M2 phenotypes were labeled with iNOS and ARG, respectively. An immunofluorescence double‐labeling experiment was carried out after microglia were stimulated with 3.2% sevoflurane for 6 h. It showed that groups with sevoflurane exposure tended to polarize into M1 phenotypes than M2 phenotypes of microglia (Figure 6A,B). The results showed that Sev and TREM1‐NC group exhibited a significant incrase in the expression of iNOS and a decrease in the expression of ARG compared with Con group. Although the expression of iNOS in the TREM1‐KD group was higher and the expression of ARG was lower than those in the Con group (*p* < 0.05), compared with the Sev group, the expression of iNOS in the TREM1‐KD group significantly decreased, and the expression of ARG increased (*p* < 0.05, Figure 6C,D).

## 4 DISCUSSION

In clinical practice, sevoflurane, a novel inhalational general anesthetic, is widely used due to its rapid induction, minimal irritation to the respiratory tract, stable anesthesia process, and quick recovery. Numerous studies have indicated its potential to cause nerve damage and progress to PNDs,^12^, ^13^ yet the specific mechanism remains unclear. In this study, sevoflurane was shown to act on its target TREM1, for the first time, to induce the inflammatory activation of microglia in vitro system. It is suggested that TREM1 may play a significant role in regulating microglial polarization under sevoflurane exposure and consequent PNDs induced by neuroinflammation.

The occurrence of PNDs is closely related to anesthesia and surgery. Our group's previous research has shown that sevoflurane exposure without surgery could induce cognitive dysfunction, accompanied by neuroinflammation and microglia activation,^14^ a finding corroborated by Zhu et al.^15^ This prompted us to identify molecular targets that interact with sevoflurane and may be involved in regulating microglial inflammatory activation. Studies reveal that under the stimulation of nervous system injury, TREM1 can upregulate the expression and synergistically amplify the inflammatory response mediated by various pattern recognition receptors, leading to uncontrolled inflammation. TREM1 activates the inflammatory corpuscles of NLRP3 and triggers microglial pyroptosis, which also plays a key role in neuroinflammation following SAH.^16^ Inhibition of TREM1 can alleviate the neurological deficit after SAH and reduce neuronal damage while simultaneously improving neuroinflammation by weakening the transformation of pro‐inflammatory microglial subtypes.^17^ Elevated TREM1 expression in the blood of patients with Alzheimer's disease (AD) is related to disease progression, dementia, and total Tau protein level, possibly linked to the immune response of AD.^18^, ^19^ Antagonizing TREM1 can inhibit the activation of microglia, reduce the levels of biomarkers and inflammatory factors related to peripheral nerve injury, and improve the motor function of mice with spinal cord injury.^20^ The above research suggests that TREM1 plays an crucial role in the occurrence and development of central nervous system diseases by enhancing the immune inflammatory reaction process of microglia. However, no direct study has examined TREM1 in PNDs induced by sevoflurane‐activated microglia, underscoring the focus of our research on assessing the role of TREM1 in sevoflurane‐induced microglial inflammatory activation. By using in vitro system, this study demonstrates, for the first time, that prolonged exposure to high concentration of sevoflurane, especially at 3.2% sevoflurane, can upregulate the expression of TREM1 in microglia, thereby inducing inflammatory activation.

Relevant local and international literature reveals a lack of specific sevoflurane concentration and intervention duration in microglia‐related research. In clinical, animal, and cell studies, the concentration of sevoflurane used is mostly between 1 and 2 minimum alveolar concentration (MAC), and the MAC of sevoflurane for tracheal intubation is 1.3 MAC. Thus, initial intervention concentrations of sevoflurane, which are commonly used in the clinic, are 2.6% (1.3 MAC), 3.2% (1.5 MAC), and 4.0% (2.0 MAC), with microglial activity and inflammatory changes detected after 6 h of intervention. The results showed that sevoflurane had little effect on the viability of microglia. The expression of TREM1 and iNOS in microglia of sevoflurane groups with different concentrations were upregulated, especially in the 3.2% sevoflurane group. ARG expression in the 3.2% sevoflurane group was significantly downregulated. By comparing the cell activity and the expression of TREM1, iNOS, and ARG at different concentrations of sevoflurane exposure, it was found that 3.2% sevoflurane exposure had the most obvious inflammatory activation on microglia. It shows that the inflammatory activation effect of sevoflurane exposure on microglia does not always increase with the increase of sevoflurane inhalation concentration. When the inhalation concentration of sevoflurane exceeds a certain range, the inflammatory activation effect of sevoflurane exposure on microglia decreases, which is consistent with Lin et al.^21^

Based on the experimental results of the effect of sevoflurane concentration on microglia, 3.2% sevoflurane was selected to intervene in microglia for different periods of time to explore the effect of sevoflurane intervention time on microglia. The intervention time selected in the experiment mimics the duration of short, medium, and long‐term sevoflurane anesthesia in the clinic and was set to 2, 4, and 6 h, respectively. Cells were collected after the intervention to detect the activity and inflammatory changes of microglia. The results indicated a negligible influence of sevoflurane intervention duration on microglial viability, with upregulation of the expression of TREM1 and iNOS, as well as downregulation of ARG expression. Notably, the 3.2% Sev 6 h group exhibited the most prominent upregulation of iNOS. It shows that the longer sevoflurane exposure time, the stronger the inflammatory activation effect on microglia, which is consistent with previous studies.^22^ Comparing the cell activity and expression of TREM1, iNOS, and ARG in each group after sevoflurane exposure for different durations, it was found that 3.2% sevoflurane exposure for 6 h had the most pronounced inflammatory activation in microglia. In summary, based on the above research findings, it is recommended that the concentration and duration of sevoflurane intervention should be set at 3.2% for 6 h.

Microglia are the major effector cells that mediate the inflammatory response of the central nervous system. When the body is stimulated or injured by the external environment, microglia can perceive different microenvironment changes and be activated and polarized into different phenotypes, namely, classical activated type (M1 type) and alternative activated type (M2 type).^23^ Among them, M1 microglia are related to the release of various pro‐inflammatory factors, such as iNOS, TNF‐α, and IL‐1β. M2 type is related to the release of various anti‐inflammatory factors, such as ARG, TGF‐β, and IL‐10.^24^, ^25^ The results of this study suggest that sevoflurane exposure can upregulate the expression of pro‐inflammatory cytokines, such as TNF‐α and IL‐1β, while knocking down TREM1 can reduce the expression of pro‐inflammatory cytokines and upregulate the expression of anti‐inflammatory cytokine‐like TGF‐β. Similarly, sevoflurane can upregulate the expression of TREM1 and iNOS, a representative factor of M1, and inhibit the expression of ARG, a representative factor of M2. Microglia are activated and polarized into M1. Knocking down TREM1 can downregulate the expression of iNOS and upregulate the expression of ARG. The results showed that TREM1 promoted microglia to increase the polarization of the M1 type and decrease the polarization of the M2 type under sevoflurane stimulation. These results showed that TREM1 plays an important role in the occurrence and development of neuroinflammation, and regulating the polarization phenotype of microglia may be one of the key factors in the treatment of sevoflurane‐induced PNDs, and TREM1 may be its target.

However, this study has some limitations. First, BV2 microglia were selected as the research object in this experiment, instead of primary microglia, to conserve animal usage and reduce frequent cell preparation. This decision was based on a study by Henn et al.,^26^ which reported that BV2 microglia were closely similar to primary mouse microglia in morphology, phenotype, and function, making them viable substitutes for primary mouse microglia for microglia‐related research. Using BV2 microglia instead of primary microglia may introduce differences in cellular behavior or responses, potentially affecting the generalizability of the findings. Second, the mechanism by which TREM1 regulates the inflammatory activation of microglia has not been extensively studied. Addressing this gap will be the focus of our future research efforts.

## 5 CONCLUSIONS

This study demonstrates that long‐term exposure to high concentrations of sevoflurane induces pronounced inflammatory activation in microglia. Moreover, TREM1 can promote the inflammatory response of microglia after long‐term high‐concentration sevoflurane intervention and regulate the polarization phenotype of microglia. These findings suggest that TREM1 could offer potential therapeutic avenues for the treatment and prevention of sevoflurane‐induced PNDs.

## AUTHOR CONTRIBUTIONS

Chunchun Tang and Zhaoqiong Zhu designed the research. Chunchun Tang performed the experiments and drafted the manuscript. Dexing Liu, Xue Zheng, Yinan Zhang, Wanqiu Yu, and Dongqin Chen participated in the experimental manipulation, collection, and assembly of data. Yuhang Zhu and Junli Jiang drawing images. All authors have read and approved the final content of this manuscript.

## CONFLICT OF INTEREST STATEMENT

The authors declare no conflict of interest.

## ETHICS STATEMENT

This study does not involve animal or human experiments and therefore does not present any ethical concerns. The cell line used in the research was purchased from Wuhan Punosai Life Science and Technology Co., Ltd. (No.: CL‐0493).

## Data Availability

Data reported in this study are available from the corresponding author on reasonable request.
